# Altermagnetism and chiral order in a collinear antiferromagnet (MnF_2_)

**DOI:** 10.1038/s41598-026-43686-3

**Published:** 2026-03-18

**Authors:** S. W. Lovesey

**Affiliations:** 1 ISIS Facility, STFC, Didcot, OX11 0QX Oxfordshire UK; 2 Diamond Light Source, Harwell Science and Innovation Campus, Didcot, OX11 0DE Oxfordshire UK; 3 Department of Physics, Oxford University, Oxford, OX1 3PU UK

**Keywords:** Materials science, Physics

## Abstract

In this work, we study hitherto unknown magnetic properties of a collinear antiferromagnet of widespread interest, because there is intense speculation that the compound is a good atomic altermagnet with spin split electronic bands (manganese fluoride, MnF_2_). The properties in question are exposed in studies of symmetry informed diffraction patterns for two standard scattering techniques. In one, the magnetically ordered solid is illuminated by x-rays tuned in energy to a Mn atomic resonance, and the radiation in the second technique is a beam of neutrons. With resonant x-ray scattering, intensity of Bragg spots are predicted to change on reversing the handedness of helicity in the primary beam. A change in intensity on switching between left and right handed x-ray circular polarization, say, equates to a magnetic chiral signature for the established magnetic space group. The same technique reveals the order parameter for altermagnetism, which is an axial magnetic octupole (third rank tensor). A multipole with identical discrete symmetries is found in the magnetic Bragg diffraction of neutrons. Moreover, spin-flip patterns from polarized neutron diffraction depend on electronic multipoles that are zero for the nominal 3d^5^ configuration of Mn^2+^, which make them excellent tests of the actual electronic structure.

## Introduction

**Fig. 1:**
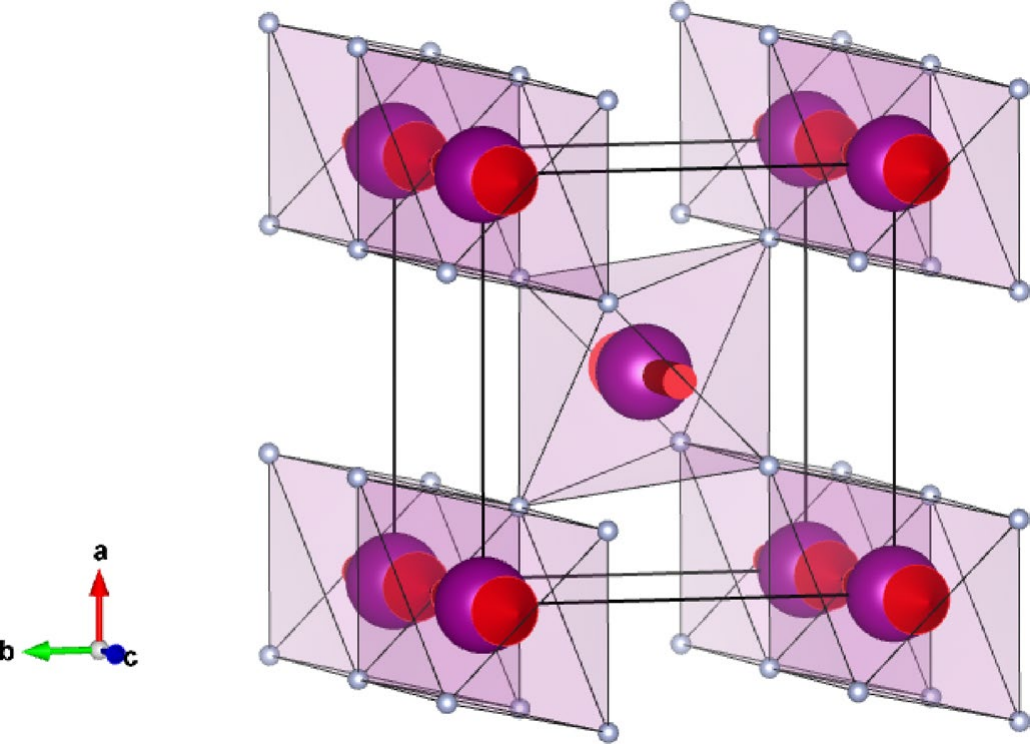
Antiferromagnetic structure of MnF_2_ including fluorine ions (reproduced from MAGNDATA, http://webbdcrista1.ehu.es/magndata). A transition metal ion is surrounded by six F^1−^ ligands forming octahedra.

Spin states in the electronic bands of an altermagnet are not degenerate, even though bulk magnetization (ferromagnetism) is forbidden by symmetry. It is a potentially useful attribute in the fabrication of next-generation devices, which accounts for a surge of interest in antiferromagnetic materials. Conditions for the desirable property are non-relativistic with no place for a spin-orbit coupling. The collinear rutile antiferromagnet MnF_2_ depicted in Fig. 1 has been promoted as an altermagnetic material^1,2^. Scattering techniques often have much to offer in gathering incisive information on properties of materials not available with other experimental methods^3^, e.g., the antiferromagnetic “hidden order parameter”. Calculation of symmetry informed Bragg diffraction patterns is a stratagem to promote this end. Using the established magnetic structure of MnF_2_, the patterns for both magnetic neutron and resonant x-ray techniques are shown to reveal the altermagnetic order parameter. Over and above this, hitherto unknown chiral magnetic order in MnF_2_ will be unveiled by resonant x-ray diffraction. Specifically, the intensity of a magnetic Bragg spot is influenced by circular polarization in the primary x-ray beam tuned in energy to a Mn atomic resonance.

Distributions of magnetization in many crystalline materials have been measured on a sub-atomic scale and compared to state-of-the-art simulations^4^. If the periodicity of the magnetic and chemical structures are the same then magnetic and nuclear scattering of neutrons occur at the same points in reciprocal space and interfere with one another. In favourable cases the polarization dependence of the interference allows accurate determinations of the magnetic amplitude. The required interference is not available in magnetoelectric solids defined by p(parity)t(time)-symmetry, because magnetic and nuclear contributions to the neutron scattering amplitude are 90^o^ out of phase^5^. In which case, magnetic and nuclear contributions to the intensity of a Bragg spot are in quadrature.

**Fig. 2:**
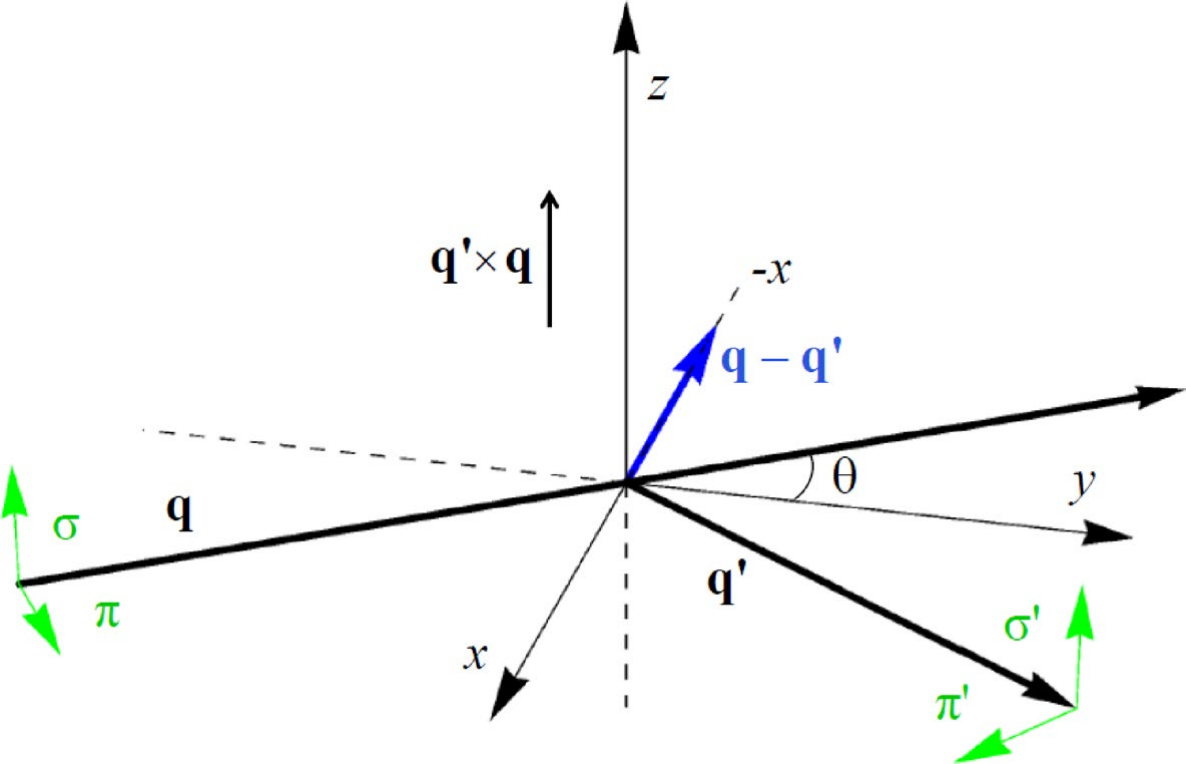
Primary (σ, π) and secondary (σ*’*, π*’*) states of polarization. Corresponding wavevectors **q** and **q***’* subtend an angle 2θ. The Bragg condition for diffraction is met when **q** − **q***’* coincides with a reflection vector (*h*, *k*, *l*) of the reciprocal lattice. Crystal vectors that define local axes (ξ, η, ζ) and the depicted Cartesian (x, y, z) coincide in the nominal setting of the crystal.

An atomic resonance in the x-ray absorption spectrum is often a sharp feature^6^. In which case, it is meaningful to assign an amplitude to the resonant contribution equal to its energy-integrated intensity. Four amplitudes are labelled by polarization states depicted in Fig. 2, and they can be developed in electronic multipoles^7^. Analytic expressions for axial multipoles for an informative atomic model are listed by Lovesey & Scagnoli^8^. In notation depicted in Fig. 2, (π’σ) denotes a rotated amplitude, and |(π’σ)|^2^ the intensity of the Bragg spot enhanced by the atomic resonance. Universal expressions for diffraction amplitudes employed here are functions of the rotation of the illuminated crystal about the reflection vector by an angle ψ^9^. Cell dimensions of MnF_2_ and the wavelengths of Mn atomic resonances mean that the Laue condition for Bragg diffraction is satisfied at the Mn K absorption edge and not at Mn L-edges.

The manganese K absorption edge has an energy E ≈ 6.552 keV (E1, 1 s → 4p). The Bragg angle in Fig. 2 is determined by (λ/2*a*), where the photon wavelength λ ≈ (12.4/E) Å, cell parameter *a* ≈ 4.8736 Å, and the x-ray energy E is in units of keV. Whence, (λ/2*a*) ≈ 0.194. The sensitivity to magnetic order at the 1 s → 4p dipole transition-energy is due to the 4p − 3 d intra-atomic Coulomb interaction and to the mixing of the 4p with the 3 d states of neighbouring magnetic ions. At the E2 threshold (E2, 1 s → 3 d) its origin is in the spin polarization of the 3 d states.

There are several previous resonant x-ray Bragg diffraction experiments relevant to our study. At room temperature haematite (α-Fe_2_O_3_) and the Mott-Hubbard compound V_2_O_3_ possess the corundum structure. Resonant x-ray Bragg diffraction by antiferromagnetic α-Fe_2_O_3_ exploiting the iron K edge (7.111 keV) is consistent with null electric dipole (E1) enhancement, and electric quadrupole (E2) azimuthal-angle scans^10^. A peculiarity of enhancement at the K edge is that spin degrees of freedom in the valence state make no contribution to electronic multipoles^11^. This means that magnetic multipoles for the ferric ion Fe^3+^ (3d^5^) available in diffraction at the K absorption edge are zero, because the nominally pure atomic state has zero orbital angular momentum. A detailed study of α-Fe_2_O_3_ showed, beyond reasonable doubt, that magnetic multipoles contribute to the diffraction pattern, a result that implies unquenched orbital angular momentum in the valence state of the material^12^. An experimental study of monoclinic antiferromagnetic V_2_O_3_ using the vanadium K edge (5.465 keV) showed strong E2 enhancement in the rotated channel of polarization^13^. Bragg diffraction studies of orthorhombic solids K_2_CrO_4_^14^ and TbMnO_3_^15^ revealed strong enhancements using a K edge absorption event (Cr K edge 5.994 keV). Azimuthal angle scans for the magnetic perovskite are consistent with null diffraction in the unrotated channel (σ’σ) and strong diffraction in the rotated channel (π’σ) of polarization and E1 enhancement^9^.

## The model

Below an ordering temperature ≈ 67 K manganese fluoride (3d^5^, Mn^2+^) is a tetragonal two-sublattice compensated antiferromagnet with Mn dipoles depicted in Fig. 1 parallel to the principal crystal axis^16^. An appreciation of altermagnetism in the past few years has propelled interest in MnF_2_ and similar compounds^2,17–19^. Turning to predictions from our symmetry informed analytic calculations, resonant x-ray and magnetic neutron diffraction should unveil the altermagnetic order parameter. In addition, resonant x-ray Bragg diffraction patterns should demonstrate that the MnF_2_ magnetic structure is chiral. Notably, the effect is forbidden, along with the piezomagnetic effect, by magnetic symmetry ($\stackrel{-}{1{\prime}}$) required for the linear magnetoelectric effect, e.g., Cr_2_O_3_^5^.

Manganese ions occupy positions (0, 0, 0) and (1/2, 1/2, 1/2) in P4_2_/mnm (No. 136) that are centres of inversion symmetry. Cell parameters are *a* = *b* ≈ 4.8736 Å, *c* ≈ 3.3000 Å^16^. Fluoride F^1−^ ions depicted in Fig. 1 are located in non-centrosymmetric positions between Mn ions.

For an atomic description of charge, orbital and spin degrees of freedom, Mn ions are assigned electronic spherical multipoles $\langle$O^K^_Q_$\rangle$ of integer rank K^7^. Projections Q are in the range − K ≤ Q ≤ + K, and angular brackets denote an expectation, or time average, value of the enclosed quantum mechanical operator. In summary, multipoles encapsulate the electronic and magnetic ground state of Mn ions in MnF_2_. Cartesian and spherical components Q = 0, ±1 of a vector **n** = (ξ, η, ζ), for example, are related by ξ = (n_−1_ − n_+1_)/√2, η = *i*(n_−1_ + n_+1_)/√2, ζ = n_0_. A complex conjugate of a multipole is defined as $\langle$O^K^_Q_$\rangle$* = [(−1)^Q^ $\langle$O^K^_−Q_$\rangle$], and the diagonal multipole $\langle$O^K^_0_$\rangle$ is purely real. The phase convention for real and imaginary parts labelled by single and double primes is $\langle$O^K^_Q_$\rangle$ = [$\langle$O^K^_Q_$\rangle$*’* + *i*$\langle$O^K^_Q_$\rangle$*’’*]. Whereupon $\langle$O^1^_ξ_$\rangle$ = −√2 $\langle$O^1^_+1_$\rangle$*’* and $\langle$O^1^_η_$\rangle$ = −√2 $\langle$O^1^_+1_$\rangle$*’’*.

The established symmetry of magnetically ordered MnF_2_ is tetragonal P4_2_*’*/mnm*’* (No. 136.499^20^,. Manganese ions in Wyckoff positions 2a form a chiral magnetic motif. Such a chiral, or handed, motif is permitted to couple to illumination that is similarly handed. Our prediction for MnF_2_ is underpinned by a symmetry informed calculation of the change in intensity of a magnetic Bragg spot on reversing the helicity (circular polarization) of the primary beam in resonant x-ray diffraction. Results for the chiral signature defined in Eq. (4) are Eqs. (5) and (7). The magnetic crystal class 4*’*/mmm*’* is centrosymmetric, permits a piezomagnetic effect, and forbids a ferromagnetic structure of axial magnetic dipoles. Wyckoff positions 2a possess oriented site symmetry m.m*’*m*’* that includes inversion symmetry. In consequence, Mn multipoles are parity even (axial). Wyckoff position symmetry includes dyad operations 2_ζ_ and 2*’*_ξη_ that demand even projections Q = 2*n*, and $\langle$O^K^_Q_$\rangle$ = [(−1)^K + *n*^ σ_θ_ $\langle$O^K^_Q_$\rangle$*], where a time signature σ_θ_ = +1 (non-magnetic, charge-like) or σ_θ_ = −1 (magnetic). However, symmetry of the unit cell forbids some multipoles appearing in Bragg diffraction patterns.

Resonant x-ray diffraction can proceed with E1-E1 (K = 0–2) or E2-E2 (K = 0–4) absorption events, for which σ_θ_ = (−1)^K^^6,7^. Whereupon the corresponding multipoles $\langle$T^K^_Q_$\rangle$ satisfy $\langle$T^K^_Q_$\rangle$ = [(−1)^*n*^ $\langle$T^K^_Q_$\rangle$*], and diagonal multipoles Q = 0 are allowed for all K, $\langle$T^K^_+2_$\rangle$ = *i*$\langle$T^K^_+2_$\rangle$*’’* and $\langle$T^K^_+4_$\rangle$ = $\langle$T^K^_+4_$\rangle$*’*. Axial magnetic multipoles in neutron diffraction $\langle$t^K^_Q_$\rangle$ are time-odd^21^. They obey $\langle$t^K^_Q_$\rangle$ = [− (−1)^K + *n*^ $\langle$t^K^_Q_$\rangle$*] for MnF_2_, meaning that $\langle$t^K^_0_$\rangle$ can be different from zero for odd K. The atomic order parameter for altermagnetism $\langle$O^3^_+2_$\rangle$ is purely imaginary, and it is visible in both resonant x-ray and magnetic neutron Bragg diffraction patterns.

## Results

A suitable electronic structure factor,

1 $${\Psi^K}_Q\left( {2a} \right){\text{ }} = \sum exp(i{{\mathbf{\kappa}}}\cdot{\mathrm{d}})\langle{O^K}_Q\rangle{_{\mathbf{d}}} = {\text{ }}[\langle{O^K}_Q\rangle + {( - 1)^h}^{ + k + l}{( - 1)^K}\langle{O^K}_Q\rangle*],$$

where the sum is over the two positions **d** used by Mn ions, and the reflection vector **κ** = (*h*, *k*, *l*) with integer Miller indices. Multipoles are set in orthonormal axes (ξ, η, ζ) that coincide with cell edges shown in Fig. 1. The result Eq. (1) follows from Wyckoff positions found in the Bilbao table MWYCKPOS for magnetic symmetry P4_2_*’*/mnm*’*^20^. Wyckoff positions are related by operations listed in the table MGENPOS^20^. Taken together, the two tables provide all information required to evaluate Eq. (1) and, thereafter, x-ray and neutron Bragg diffraction patterns. The reflection condition even (*h* + *k* + *l*) for nuclear scattering follows from the requirement that Ψ^K^_0_(2a) is different from zero for even K. Fluorine ions in Wyckoff positions 4f do not diffract at (*h*, 0, *l*) with odd (*h* + *l*), and these purely magnetic reflections are the subject of the present study of resonant x-ray Bragg diffraction. It follows that,

2 $${\Psi^K}_Q\left( {2a} \right){\text{ }} = \langle{T^K}_Q\rangle[1 - {( - 1)^{K + n}}],$$

with Q = 2*n*. Specifically, Ψ^K^_0_(2a) = 0, Ψ^K^_+2_(2a) = 2*i* $\langle$T^K^_+2_$\rangle$*’’*, Ψ^K^_+4_(2a) = 0 for even K, and Ψ^K^_0_(2a) = 2$\langle$T^K^_0_$\rangle$, Ψ^K^_+2_(2a) = 0, Ψ^K^_+4_(2a) = 2 $\langle$T^K^_+4_$\rangle$*’* for odd K. Magnetic dipoles $\langle$T^1^_0_$\rangle$ are parallel to the c-crystal axis as in Fig. 1.

Diffraction amplitudes have been calculated with Ψ^K^_Q_(2a) in Eq. (2) and universal expressions provided by Scagnoli & Lovesey^9^. Using an E1-E1 absorption event, and notation cos(χ) = (*h*/*a*) [(*h*/*a*)^2^ + (*l*/*c*)^2^]^−1/2^ we find,

3 $$\begin{aligned}&(\sigma^\prime\sigma ){\text{ }} = - sin(\chi ){\text{ }}sin(2\psi )\left\langle {{T^2}_{+2}}\right\rangle^{\prime\prime},\\& (\pi^\prime\sigma ){\text{ }} = - cos(\chi ){\text{ }}cos(\theta ){\text{ }}sin(\psi ){\text{ }}[(i/\surd 2)\left\langle {{T^1}_0} \right\rangle + \left\langle {{T^2}_{ + 2}} \right\rangle^{\prime\prime}]\\&\qquad\qquad\qquad + sin(\chi ){\text{ }}sin(\theta ){\text{ }}[(i/\surd 2)\left\langle {{T^1}_0} \right\rangle - cos(2\psi )\left\langle {{T^2}_{ + 2}} \right\rangle ''].\end{aligned}$$

Referring to Fig. 2, for the azimuthal angle ψ = 0 the η and y axes are antiparallel. The quadrupole $\langle$T^2^_+2_$\rangle$*’’* contributes Templeton-Tempelton (T & T) scattering^22^. Notably, (σ*’*σ) is purely real and (π*’*σ) is complex. These features imply a non-zero chiral signature taken up in the next section.

## Chiral signatures

Intensity of a Bragg spot picked out by circular polarization in the primary photon beam equals P_2_ϒ^14^ with,

4 $$\Upsilon={\text{ }}\{ (\sigma^\prime \pi)^*(\sigma^\prime\sigma)+(\pi^\prime \pi)^*(\pi^\prime \sigma)\}^{\prime\prime},$$

and a Stokes parameter P_2_^7^ measures helicity in the primary x-ray beam. Intensity is a scalar quantity, and ϒ and P_2_ possess identical discrete symmetries, namely, both scalars are time-even and parity-odd. Partial intensity P_2_ϒ different from zero is a signature of diffraction by a chiral magnetic symmetry, of course. Evaluation of the chiral signature ϒ demands a knowledge of all four diffraction amplitudes. For forbidden reflections (2*m* + 1, 0, 0) and an E1-E1 absorption event,

$$(\sigma^{\prime}\sigma )\, = \,0,{\text{ }}(\pi^{\prime}\pi )\, = \,i\:sin(2\theta ){\text{ }}cos(\psi )\left\langle {{T^1}_0} \right\rangle ,{\text{ }}(\pi ^{\prime}\sigma ){\text{ }} = - cos(\theta ){\text{ }}sin(\psi ){\text{ }}[(i/\surd 2)\left\langle {{T^1}_0} \right\rangle + \left\langle {{T^2}_{ + 2}} \right\rangle^{\prime\prime}],$$

5 $$\Upsilon \left( {E1 - E1} \right)\, = \,sin(2\theta ){\text{ }}cos(\theta ){\text{ }}sin(2\psi )\left\langle {{T^1}_0} \right\rangle \left\langle {{T^2}_{ + 2}} \right\rangle^{\prime\prime}.$$

The crystal c axis is normal to the plane of scattering for ψ = 0. The chiral signature is created by inference between a magnetic dipole and T & T scattering. However, this is not a general result, e.g., the chiral signature for a Sohncke-type magnetic material Pb(TiO)Cu_4_(PO_4_)_4_ includes a product of dipoles^23^. Notably, there is no E1-E1 scattering in the unrotated channel (σ*’*σ), which simplifies the calculation of the chiral signature. Results here for (σ*’*σ) and (π*’*σ) follow from Eq. (3) on setting the Miller index *l* = 0, and sin(χ) = 0. Amplitudes for an E2-E2 event are,

6 $$(\sigma^{\prime} \sigma )\, = \,i\: sin(2\theta ){\text{ }}cos(\psi ){\text{ }}[ - \left\langle {{T^1}_0} \right\rangle + \{ 5{\text{ }}co{s^2}(\psi ) - 3\} \left\langle {{T^3}_0} \right\rangle ],$$

$$\begin{aligned}(\pi '\sigma )\, = &\,isin(\psi ){\text{ }}[cos(3\theta )\left\langle {{T^1}_0} \right\rangle + \{ 3{\text{ }}cos(3\theta ) - 5{\text{ }}cos(\theta ){\text{ }}(3{\text{ }}co{s^2}(\theta ) - 2){\text{ }}si{n^2}(\psi )\} \left\langle {{T^3}_0} \right\rangle \\&- i\surd \left( {30/7} \right){\text{ }}Z],\end{aligned}$$

with a purely real sum of non-magnetic (T & T) multipoles,

$$Z{\text{ }} = {\text{ }}[cos(3\theta )\left\langle {{T^2}_{ + 2}} \right\rangle^{\prime\prime} + (1/\surd 3){\text{ }}cos(\theta ){\text{ }}\{ (7{\text{ }}co{s^2}(\psi ) - 1){\text{ }}co{s^2}(\theta ) - 1\} \left\langle {{T^4}_{ + 2}} \right\rangle^{\prime\prime}].$$

Leading to a chiral signature,

7 $$\Upsilon \left( {E2 - E2} \right)\, = \,sin(2\theta ){\text{ }}sin(2\psi ){\text{ }}Z{\text{ }}[\{ 8{\text{ }}co{s^2}(\theta ) - 5\} \left\langle {{T^1}_0} \right\rangle + co{s^2}(\theta ){\text{ }}\{ 5{\text{ }}co{s^2}(\psi ) - 3\} \left\langle {{T^3}_0} \right\rangle ].$$

Both chiral signatures are odd with respect to the azimuthal angle, and functions of (2ψ). Manganese ions will possess unquenched orbital angular momentum if $\langle$T^3^_0_$\rangle$ is different from zero at the K edge, which has been established for ferric ions in haematite^12^.

## Neutron polarization analysis

A dependence of the magnetic neutron scattering amplitude on both the magnitude and direction of the reflection vector **κ** is a most valuable property of the technique. It enables the measurement of the magnetization density, or its spatial Fourier transform more correctly, and the identification of electron back-transfer (covalency)^4^. Specifically, multipoles in neutron diffraction are strong functions of the magnitude of the reflection vector. This feature is illustrated in a useful approximation to the axial dipole $\langle$**t**^1^$\rangle$,

8 $$\left\langle {{{\mathbf{t}}^1}} \right\rangle \approx (\left\langle {{\mu}} \right\rangle /3){\text{ }}[\left\langle {{j_0}(k)} \right\rangle + \left\langle {{j_2}(k)} \right\rangle ({g_o} - 2)/{g_o}].$$

The quantities $\langle$j_0_(*κ*)$\rangle$ and $\langle$j_2_(*κ*)$\rangle$ are spherical Bessel functions averaged over the radial distribution of electrons in the valence state. In the forward direction of scattering $\langle$j_0_(0)$\rangle$ = 1 and $\langle$j_2_(0)$\rangle$ = 0, and the principal maximum of $\langle$j_2_(*κ*)$\rangle$ occurs at *κ* ≈ 6.3 Å^−1^ ^24^. Neutron Bragg diffraction by ordered MnF_2_ is presented by Alperin et al.^25^. and Yamani et al.^16^. Returning to Eq. (8), the magnetic moment $\langle$**µ**$\rangle$ = g_o_ $\langle$**S**$\rangle$ and the orbital moment $\langle$**L**$\rangle$ = [(g_o_ − 2) $\langle$**S**$\rangle$]. The coefficient of $\langle$**L**$\rangle$ is approximate, while $\langle$**t**^1^$\rangle$ = (1/3) $\langle$2**S** + **L**$\rangle$ for *κ* → 0 is an exact result. Higher order multipoles with even rank depend on the electronic position operator **n**. The equivalent operator [$\langle$j_2_(*κ*)$\rangle$ (**S × n**) **n**] for **t**^2^ shows that the quadrupole measures the correlation between the spin anapole (**S × n**) and orbital degrees of freedom^21^. Magnetic neutron multipoles with an even rank do not exist for magnetic states derived from a state specified by one value of the total angular momentum J. Instead, a ground state must possess two, or more, J-states before $\langle$**t**^2^$\rangle$ is non-zero^21^. A single J-state is at odds with the basic premise of altermagnetism, because the singular state is one outcome of strong spin-orbit coupling. Moreover, higher order multipoles are zero for the nominal atomic state Mn^2+^ (3d^5^)^21^. Evidence to the contrary in MnF_2_ can be obtained from Bragg spots at overlapping nuclear-magnetic reflections indexed by even (*h* + *k* + *l*).

Intensity of a magnetic Bragg spot = |$$\langle$$**Q**$$_\bot$$$$\rangle$$|^2^, where $$\langle$$**Q**$$_\bot$$$$\rangle$$ = [**e** $$\times$$ ($$\langle$$**Q**$$\rangle$$ $$\times$$ **e**)], the unit vector **e** = **κ**/*κ*, and the neutron scattering amplitude $$\langle$$**Q**$$\rangle$$ is a sum of spin and orbital magnetizations illustrated with the dipole approximation in Eq. (8)^21^. A fraction $$\propto$$ {(1/2) (1 + P^2^) |$$\langle$$**Q**$$_\bot$$$$\rangle$$|^2^ − |**P** ∙ $$\langle$$**Q**$$_\bot$$$$\rangle$$|^2^} of neutrons participate in events that change (flip) the neutron spin orientation, where **P** is the primary polarization. An assumption of perfect polarization (**P** ∙ **P**) = 1 yields a standard spin-flip signal^26^,

9 $$SF{\text{ }} = {\text{ }}[|{{\mathbf{Q}}_ \bot }{|^2} - |{\mathbf{P}}\cdot\left\langle {{{\mathbf{Q}}_ \bot }} \right\rangle {|^2}].$$

Evidently, all scattering is spin-flip when **P** and **e** are aligned since **e** ∙ $$\langle$$**Q**$$_\bot$$$$\rangle$$ = 0. Amplitudes for MnF_2_ correct to the level of magnetic octupoles, and even (*h* + *k* + *l*) are^21^,

10 $$\left\langle {{Q_\xi }} \right\rangle \approx ({e_\eta }{e_\zeta })f,\left\langle {{Q_\eta }} \right\rangle \approx ({e_\zeta }{e_\xi })f,\left\langle {{Q_\zeta }} \right\rangle \approx ({e_\xi }{e_\eta })g,$$

$$f = \langle{t^2}_{+2}\rangle^{\prime} + (1/2)\surd (35/2)\langle{t^3}_{+2}\rangle^{\prime\prime},{\text{ }}(2f + g){\text{ }} = {\text{ }}(3/2)\surd (35/2)\langle{t^3}_{+2}\rangle^{\prime\prime}.$$

The octupole $\langle$t^3^_+2_$\rangle$*’’* measures a bulk magnetic octupole in MnF_2_. On choosing e_η_ = 0 we note 2(e_ζ_ e_ξ_) = sin(2χ), where χ is an angle in Eq. (3). Standard choices for the neutron polarization include (a) **P** and **e** parallel (b) **e** ∙ *P* = 0 using **P** = (− e_ζ_, 0, e_ξ_), and (c) **e** ∙ *P* = 0 using **P** = (0, 1, 0). The corresponding spin-flip signals are SF(a) = [(e_ζ_ e_ξ_) ƒ]^2^, SF(b) = SF(a), and SF(c) = 0. Thus, diffraction conditions (a) and (b) access $\langle$t^3^_+2_$\rangle$*’’*.

## Atomic altermagnetism

The well-established Rashba-Dresselhaus effect removes the spin degeneracy of electronic bands for material conditions unlike altermagnetism^27–29^. It applies to nonmagnetic materials with finite spin-orbit coupling, and the absence of a centre of inversion symmetry, e.g., an acentric crystal structure. By elaborating a theoretical concept put forward by Peka & Rashba^30^, Yuan et al.^17^. demonstrate a spin-splitting that depends on the electron band momentum, and it operates even in centrosymmetric antiferromagnets. Most of the 69 altermagnetic crystal classes permit the piezomagnetic effect (permitted in 66 of the total 90 magnetic crystal classes), and they include collinear antiferromagnets^18^. Crucially, ferro-type ordering of magnetic axial multipoles with rank K > 1 is permitted, even though axial magnetic dipoles (K = 1) form a fully compensated antiferromagnetic structure. Other potentially useful material properties, all accomplished without ferromagnetism, are efficient spin-current generation, spin-splitting torque, giant magnetoresistance, and an anomalous Hall effect.

Manganese fluoride is an atomic altermagnetic with ferroically ordered axial magnetic octupoles, i.e., bulk octupole magnetism^19,31^. They are denoted here by $\langle$**T**^3^$\rangle$ and $\langle$**t**^3^$\rangle$ in resonant x-ray and magnetic neutron diffraction, respectively. Our electronic structure factor Eq. (1) with Miller indices *h* = *k* = *l* = 0 delineates bulk properties of symmetry P4_2_*’*/mnm*’* (No. 136.499), specifically, Ψ^3^_Q_(2a) = 2*i* $\langle$t^3^_Q_$\rangle$*’’* for neutrons. In the application of symmetry for Wyckoff positions 2a we use σ_θ_ = −1, which is correct for magnetic neutron diffraction and odd rank $\langle$**T**^K^$\rangle$. Continuing with a calculation of $\langle$t^3^_Q_$\rangle$*’’*, position symmetry shows that it can be different from zero for projections Q = ± 2. An E2-E2 absorption event accesses the altermagnetic order parameter $\langle$T^3^_+2_$\rangle$*’’*. It is invisible in purely magnetic reflections, however. Using a reflection (2*m*, 0, 0),

$$(\pi^{\prime}\sigma )\, = \,sin(2\psi ){\text{ }}[sin(3\theta )\left\langle {{T^2}_0} \right\rangle + sin(\theta ){\text{ }}\{ [(\surd 5/3){\text{ }}(4{\text{ }}co{s^2}(\theta ) - 1 - 7{\text{ }}{(cos(\theta ){\text{ }}sin(\psi ))^2})]\left\langle {{T^4}_0} \right\rangle$$

11 $$+ (\surd 14/3){\text{ }}[1 - {(cos(\theta ){\text{ }}sin(\psi ))^2}]\left\langle {{T^4}_{ + 4}} \right\rangle^\prime\} - i\surd \left( {7/6} \right){\text{ }}\{ sin(\theta ) + sin(3\theta )\} \left\langle {{T^3}_{ + 2}} \right\rangle^{\prime\prime}\} ].$$

Order parameter and T & T contributions to the E2-E2 amplitude are 90^o^ out of phase, and they are in quadrature in the Bragg spot intensity |(π*’*σ)|^2^. The phase shift between magnetic $\langle$T^3^_+2_$\rangle$*’’* and nonmagnetic T & T contributions we find in the MnF_2_ amplitude for diffraction at a space group allowed reflection is the analogue of the the phase shift between magnetic and nuclear contributions to the neutron diffraction amplitude^25^. Intensity is a four-fold periodic function of the azimuthal angle. At the origin of a scan in ψ the crystal c axis is normal to the plane of scattering in Fig. 2.

## Conclusions and discussion

In summary, we demonstrate that the established magnetic symmetry of MnF_2_ is chiral. To this end, we made a symmetry informed calculation of magnetic Bragg spots including circular polarization in a primary beam of x-rays tuned in energy to a manganese atomic resonance. Specifically, non-magnetic (time-even and charge like) and magnetic (time-odd) contributions to the four diffraction amplitudes, labelled by four states of photon polarization in Fig. 2, are not of one phase. This finding is correct for axial electric dipole (E1-E1) and electric quadrupole (E2-E2) diffraction amplitudes. Polar E1-E2 amplitudes are forbidden by inversion symmetry in the Wyckoff positions used by Mn ions. By contrast, parity-time symmetry in the linear magnetoelectric effect imposes identical phases on time-even and time-odd contributions to the four amplitudes, and the intensity of a Bragg spot is immune to circular polarization in the primary beam of x-rays. Our scattering amplitudes and chiral signatures are functions of an azimuthal angle that measures rotation of the MnF_2_ crystal about the reflection vector.

A study of polarization analysis of magnetic neutron diffraction by MnF_2_ complements results for resonant x-ray Bragg diffraction. The emphasis is on magnetic quadrupoles and octupoles that contribute to mixed nuclear-magnetic Bragg spots. Since they are zero for a nominal electronic configuration Mn^2+^ (3d^5^) there is potential in future experiments to reveal subtle effects in the electronic structure.

The altermagnetic order parameter is exposed in both magnetic neutron and resonant x-ray Bragg diffraction patterns. In the latter case it appears in space group allowed Bragg spots with magnetic and nonmagnetic contributions 90^o^ out of phase, which is anticipated from earlier studies of magnetic neutron diffraction by MnF_2_.

The stratagem that reveals altermagnetism and chiral order in MnF_2_ has been applied to candidate materials RuO_2_ and MnTe^32,33^. Absence of magnetic order in bulk RuO_2_ has been established beyond reasonable doubt by a number of researchers using various experimental techniques^34–37^. Attention has turned to two MnTe candidates that belong to magnetic crystal classes mmm or m′m′m^38–40^. Their Néel vectors enclose an angle of 30^o^. The piezomagnetic effect has opposite signs for mmm and m′m′m but currently available measurements are not conclusive^38^. In the case of mmm, an altermagnetic order parameter is exposed in both magnetic neutron and resonant x-ray Bragg diffraction patterns.

We note by the by that magnetic neutron and x-ray scattering amplitudes are similar in non-resonant scattering. Commonality occurs in the first correction to the Thomson scattering length in an expansion in (E/m_e_c^2^), where E is the primary energy and the rest mass of an electron (m_e_c^2^) = 0.511 MeV^7^. De Bergevin and Brunel made the first report of magnetic photon scattering in 1972 in the diffraction of CuKα radiation by a single crystal of NiO^41^. Subsequent studies exploited very hard x-rays (80 keV-100 keV) from synchrotron sources to contrast surface and bulk magnetism, e.g., MnF_2_^42^. Another advantage of x-ray over neutron magnetic scattering is that magnetic dipoles, spin and orbital moments, can be separately measured. Likewise, identical higher-order multipoles in the two scattering techniques are present under different conditions^7^.

## Data Availability

The data sets used and/or analyzed during the current study are available from the corresponding author upon reasonable request.
